# The Effects of Breeding Methods on Cecal Microflora and Production Traits of Yimeng Black Goats

**DOI:** 10.3390/ani16081156

**Published:** 2026-04-10

**Authors:** Yan Yang, Fukuan Li, Chenhong Zhang, Fuxia Li, Meiying Song, Shenjin Lv, Zhennan Wang

**Affiliations:** 1 Linyi Academy of Agricultural Science, Linyi 276000, China; yangyan_lv@163.com (Y.Y.); lifuxia89@163.com (F.L.); songmy089@163.com (M.S.); 2 College of Agriculture and Forestry Sciences, Linyi University, Linyi 276000, China; lifukuan@lyu.edu.cn (F.L.); m15726006117@163.com (C.Z.)

**Keywords:** breeding method, cecum, microbiota, Yimeng Black goats, 16S rRNA

## Abstract

This study investigated the effects of different breeding methods on the cecal microbiota and production traits of Yimeng Black Goats (YBGs). Twenty-seven 3-month-old male YBGs were assigned to three groups (*n* = 9 each): total mixed ration once daily (A), concentrate in the morning and roughage in the afternoon (B), or grazing with supplementary feeding (C). Cecal bacterial communities were analyzed via 16S rRNA sequencing, and functional potential was predicted using FAPROTAX. Breeding method significantly altered microbial composition (*p* < 0.05). Beta diversity was highest in Group C, while alpha diversity remained similar across groups. *Bacteroidetes*, *Firmicutes*, and *Proteobacteria* were dominant; *Proteobacteria* were most abundant in Group A. At the genus level, relative abundances of nine taxa, including *Lactobacillus* and *Fusobacterium*, differed significantly (*p* < 0.05). At the species level, including *Lactobacillus mucosae*, *Bacteroides massiliensis* and *Alistipes finegoldii*, differed significantly (*p* < 0.05). Chemoheterotrophy and fermentation functions were most enriched, particularly in Group C. Total weight gain was highest in Group A and lowest in Group C (*p* < 0.05), while carcass rate showed no significant differences (*p* > 0.05). Correlation analysis revealed that *Proteobacteria*, *Fusobacteria*, and *Euryarchaeota* as the key phylum, and *Bacteroides*, *Tyzzerella*, *Fusobacterium*, *unidentified_Prevotellaceae*, *Methanovrevibacter* and *Faecalibacterium* as the key genera were influencing the production traits of YBGs. These findings highlight the adaptive responses of the cecal microbiota to breeding methods and their potential links to host performance.

## Simple Summary

This study examined how different feeding methods affect the cecum bacteria and growth performance of Yimeng Black Goats. Twenty-seven goats were divided into three groups: total mixed ration, separate feeding of concentrate and roughage, and grazing with supplementation. Results showed that breeding methods significantly altered the composition of cecal microbiota, with notable differences in bacterial diversity and abundance at various taxonomic levels. Grazing goats had higher microbial diversity and functional enrichment for chemoheterotrophy and fermentation. Total weight gain was highest in goats fed a total mixed ration, while carcass rate remained unchanged across groups. Key bacterial groups were identified as potentially influencing production traits.

## 1. Introduction

Mutton and chevon have been gaining prominence in the human diet, driven by a growing global population and increasing wealth. Yimeng Black goats (YBGs), as a native breed, are mainly raised in central and southern Shandong Province, China. It is highly favored by people, and it is renowned for its meat, fur and wool. They possess notable advantages such as rough feeding tolerance, strong adaptability and good meat quality. However, traditional management practices like grazing, coupled with inherent traits such as seasonal estrus and low lambing percentages, have resulted in disadvantages including poor nutrition utilization, slow growth, and low production efficiency [1]. Consequently, improving growth performance is crucial for the YBG industry [2]. Currently, intensive and efficient livestock farming development, transitioning from grazing to house feeding, has become a common strategy to improve the growth performance [3,4]. Wang et al. [5] and Ji et al. [6] all found that house feeding led to significant improvements in both growth and slaughter performance of Tibetan sheep compared to grazing. The above facilitated the transformation of YBGs from grazing to house feeding.

Furthermore, within housed systems, implementing appropriate nutritional management, such as optimizing concentrate-to-roughage ratios and utilizing Total Mixed Rations (TMR), could significantly enhance overall health and growth performance of livestock [7]. Liu et al. [8] indicated that increasing the dietary concentrate content improved the growth performance of Tibetan sheep, with those fed a 45% concentrate diet achieving the best results. However, the feeding modes in which the same amount of feed is distributed throughout the day can also have an impact on animals. Zhang et al. [9] found that feeding diets with a high-concentrate ratio during the day significantly reduced rumen pH, increased NH_3_-N concentration and the activities of amylase and lipase, and improved the microbial diversity. Moreover, the total mixed ration (TMR) feeding mode is widely used in ruminant production [10]. Bargo et al. [11] found that TMR feeding induced higher milk production, as well as higher milk fat and true protein percentages, in dairy cows compared with sequential feeding of concentrate and roughage. However, Șanta et al. [12] reported that cows fed a TMR diet yielded a higher quantity of milk, but exhibited lower milk fat and protein contents, as well as higher concentrations of saturated fatty acids and n-6 fatty acids, along with a lower content of lipophilic antioxidants in milk, compared with those fed a grazing plus partial mixed diet. Therefore, the breeding methods in housed systems warrant further investigation, particularly in the YBGs, which remain poorly studied.

The gastrointestinal tract comprises the digestive and absorptive organs of animals. Gastrointestinal microorganisms play a crucial role in the normal development, digestion, and metabolism of the host [13]. Their composition and functions were altered by the feeding pattern [14], and some of them are significantly associated with animal growth performance. Wang et al. [15] reported a negative correlation between rumen *Prevotella* and the average daily gain of *Hu* sheep. Moreover, Yang et al. [16] identified *Muribaculaceae* and *Oscillospiraceae* in the rumen, rectum, and colon as key microbial taxa influencing growth performance and immunity in *Hu* sheep. Despite these insights, existing research on ruminant gastrointestinal microorganisms has largely focused on the rumen and feces [17,18,19], overlooking other critical regions such as the cecum. Cecum serves as a major fermentation region in hindgut fermenters, where microorganisms further degrade chyme incompletely fermented in the rumen [9]. Given its crucial role in nutrient utilization and energy harvest, the cecal microbiota may play a pivotal, yet underappreciated, role in regulating host growth performance and fat deposition [20]. Consequently, how different housed feeding strategies influence this specific microbial niche, and how these microbial shifts subsequently translate into variations in production traits in goat breeds like the YBGs, remains a critical knowledge gap.

Based on the above, we hypothesized that different housed breeding systems (total mixed ration versus a conventional separate feeding regimen) or grazing systems would not only shape distinct cecal microbial communities in YBGs but also establish unique host–microbe interactions that directly correlate with variations in their production traits. Therefore, the primary objectives of this study were: (1) to investigate the effects of distinct breeding methods on the production traits and cecal microflora of YBGs; and (2) to identify key cecal microbial taxa associated with these production traits through correlation analysis. This study moves beyond simply confirming the superiority of housed feeding over grazing. Instead, it seeks to provide a mechanistic understanding of how cecal fermentation, mediated by the cecal microbiota, contributes to growth performance in a local goat breed undergoing a critical management transition. The findings are expected to offer a theoretical basis and a practical reference for refining feeding strategies in housed systems, ultimately promoting sustainable and efficient production in the YBG industry.

## 2. Materials and Methods

### 2.1. Sample Acquisition

The trial was conducted in the Yinan Yimeng Black goat breeding cooperative, Linyi, China (118.34357° E, 35.3981° N). The site features a temperate monsoon climate and a semi-intensive grazing system typical for YBGs in the region. The cooperative maintained a flock of approximately 600 goats under consistent husbandry practices, with grazing on native shrub–grass vegetation and supplemental feeding. This setting provided representative conditions for evaluating breeding methods under local production systems.

Twenty-seven 3-month-old male YBGs weighing about 8.1 kg on average were selected from the cooperative’s flock for this study. All 27 goats were born within a 10-day period and were offspring of multiparous does from the same breeding group, ensuring a similar genetic background. Goats were weaned at 60 days of age and had been raised under identical management conditions prior to the trial. All goats were clinically healthy, had completed routine vaccination, and had been dewormed two weeks prior to the trial. The goats were then randomly and uniformly assigned to three treatment groups (*n* = 9 per group). Goats in Group A were fed a TMR once daily at 7:00 a.m. Goats in Group B were fed concentrate at 7:00 a.m., and roughage at 5:00 p.m. Goats in Group C were grazed from 8:30 a.m. to 4:30 p.m., followed by a supplementary fodder of 375 g/d. The main nutrient contents of TMR, concentrate, roughage and supplementary fodder were shown in Table 1. All goats were housed in individual pens, including supplementary feeding and rest period for goats in Groups C. Each pen was equipped with an individual feeder and an automatic waterer, allowing ad libitum access to clean water. Health status was monitored twice daily at 6:30 am and 18:00 pm throughout the experiment, and no health issues were observed.

The experiment commenced on 8 August 2018, comprising a 15-day adaptation period and a 60-day formal trial period. Each group contained 9 replicates. During the 15-day adaptation period, all goats were gradually transitioned to their respective dietary treatments to minimize digestive disturbances. In the adaptation period, goats acclimated to feeding management protocols. On both the first and last days of the formal trial, the goats were weighed prior to the morning feeding (12 h fasting, but water ad libitum) to calculate the total weight gain (TWG). Goats were weighed individually using a calibrated electronic livestock scale (accuracy ± 0.1 kg, Shanghai Pingxuan Scientific Instrument Co., Ltd., Shanghai, China).

On the terminal day of the trial, all goats were slaughtered. Carcass weight (CW) was immediately measured. Meanwhile, cecal digesta from the intermediate region area was collected and homogenized into 50 mL centrifuge tubes. The tubes were placed immediately in liquid nitrogen and then stored in an ultra-low temperature freezer at −80 °C until the determination of cecal microflora to capture the cumulative effect of 60-day breeding methods.

### 2.2. DNA Extraction

Total genomic DNA was extracted from cecal digesta samples using the cetyltriethylammonium bromide (CTAB) method as previously described [21]. DNA concentration and purity were assessed by electrophoresis on 1% agarose gels. Based on spectrophotometric quantification, DNA was diluted to 1 ng/μL using sterile water for downstream applications.

### 2.3. 16S rRNA Amplification and Sequencing

The V3–V4 hypervariable region of the bacterial 16S rRNA genes was amplified using the specific primer pair 341F (CCTACGGGRBGCASCAG) and 806R (GGACTACNNGGGTATCTAAT) by PCR. PCR products were mixed with an equal volume of 1X loading buffer containing SYBR Green and subsequently separated by electrophoresis 2% agarose gels for quality assessment. Amplicons were pooled in equimolar ratios based on band intensity quantification, followed by purification using the Qiagen Gel Extraction Kit (Qiagen, Hilden, Germany). Sequencing libraries were constructed using the Illumina TruSeq DNA Kit (Illumina, San Diego, CA, USA) following the manufacturer’s instructions. Library quality was assessed via the Qubit@ 2.0 Fluorometer (Thermo Scientific, Carlsbad, CA, USA) for DNA quantification and the Agilent Bioanalyzer 2100 system (Agilent Technologies, Inc., Palo Alto, CA, USA) for fragment size distribution. Final libraries were sequenced on an Illumina NovaSeq platform, generating 250 bp paired-end reads [21].

### 2.4. Bioinformatics and Statistical Analysis

Raw tags were filtered using QIIME (V1.9.1) to perform high-quality clean tags. And based on the reference database (Silva database) [22], mismatched chimera sequences were detected and removed. Sequences with ≥97% similarity were clustered into OTUs. Representative OTU sequences were screened for species annotation and phylogenetic relationship construction. Species annotation analysis was conducted using the Mothur method and the SILVA132 (http://www.arb-silva.de/) [23] SSUrRNA database [24] (with a threshold set at 0.8 to 1), to obtain taxonomic information and to statistically analyze the community composition of each sample at various taxonomic levels: phylum, genus, and species. Sequencing depths were evaluated by the rank-abundance and rarefaction curves. The relative abundance of each sample was normalized using feature abundance. And the alpha and beta diversities were analyzed by QIIME (V1.9.1).

To assess sequencing depth adequacy, rarefaction curves and rank abundance curves were generated. For diversity analyses, samples were rarefied to an even sequencing depth to normalize for variations in sequencing depth. Alpha diversity indices, including the Chao1 richness estimator, observed OTUs, Goods coverage, the Shannon diversity index, and the Simpson diversity index, were calculated using QIIME (V1.9.1). Beta diversity was assessed using weighted and unweighted UniFrac distances, and community structure differences were visualized using principal coordinate analysis (PCoA), principal component analysis (PCA), and nonmetric multidimensional scaling (NMDS) based on the UniFrac distance matrices.

### 2.5. Statistical Analysis

All statistical analyses were performed using R (v4.1.0), SPSS 27 and QIIME (V1.9.1). One-way analysis of variance (ANOVA) followed by Duncan’s post hoc test was used to compare alpha diversity indices and production traits among three groups, with statistical significance set at *p* < 0.05. Permutational multivariate analysis of variance (PERMANOVA) with permutations was applied to test for significant differences in beta diversity among groups. Differentially abundant taxa were identified using linear discriminant analysis effect size (LEfSe) with a logarithmic LDA score threshold > 4.0. Pairwise comparisons of specific taxa between groups were performed using an unpaired two-tailed Student’s *t*-test. Spearman’s rank correlation coefficient was employed to assess associations between the relative abundances of cecal microorganisms and production traits, with no adjustment for multiple comparisons, given the exploratory nature of the analysis.

The sample size (*n* = 9 per group) was determined by the availability of uniform animals from the cooperative’s flock. Although the sample size is modest, the observed differences in total weight gain were substantial. Post hoc effect size analysis revealed Cohen’s d values of 7.26 (A vs. B), 11.07 (A vs. C), and 6.52 (B vs. C), indicating very large effect sizes according to Cohen’s criteria [25]. These large effect sizes, combined with highly significant *p*-values (*p* < 0.001), demonstrate that the treatment effects are biologically meaningful and detectable despite the limited sample size. All data are presented as means ± standard deviation (SD). Statistical significance was considered at *p* < 0.05.

## 3. Results

### 3.1. Sequencing Results and Bacterial Diversity

**Figure 1:**
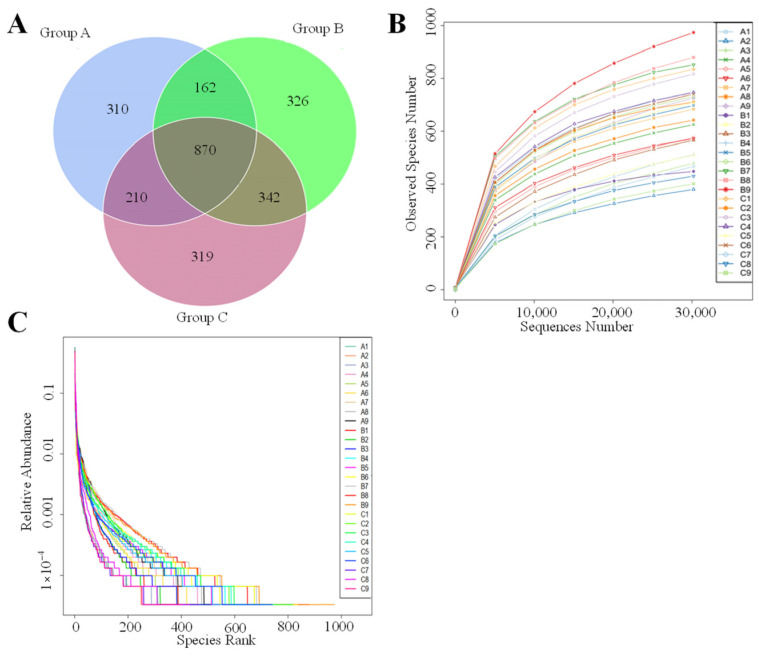
Sequencing results. (**A**) The Venn diagram shows three groups of the OTUs distribution; (**B**) rarefaction curve for all samples; (**C**) rank abundance for all samples. A1 to A9 represent the sample in Group A. The same as B1 to B9 and C1 to C9.

Following taxonomic classification, a total of 5014 OTUs were identified across all samples, with 870 OTUs shared among them, representing approximately 17.35% of the total OTUs. Specifically, the cecal microbiota of Group A, Group B and Group C contained 1556, 1721 and 1737 OTUs, respectively (Figure 1A). And their specific OTUs were 310, 326, and 319, respectively (Figure 1A). The species accumulation curve reached a plateau when the number of effective sequences exceeded 30,000, indicating that the sequencing depth and quantity meet the requirements for reliable analysis (Figure 1B). Moreover, the rank abundance curves for all samples exhibited broad distributions with gentle declines, indicating high evenness and richness (Figure 1C).

**Figure 2:**
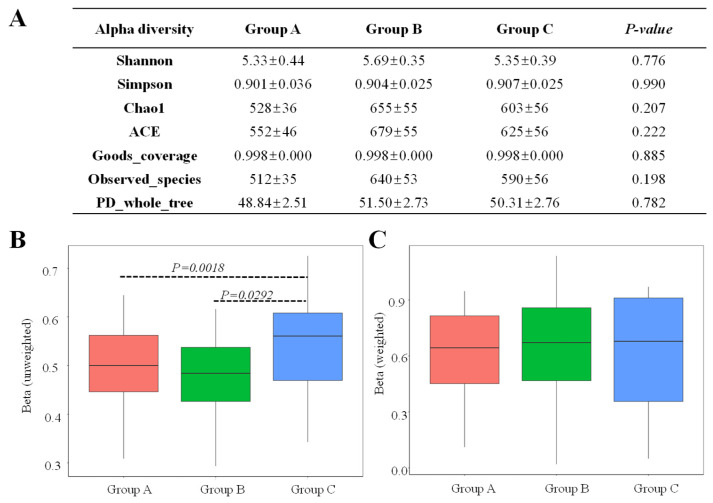
Statistical analysis of alpha and beta diversity. (**A**) Effect of three breeding methods on microbial alpha diversity; (**B**,**C**) indicated beta diversity on the basis of the unweighted and weighted UniFrac distance. Significant differences were shown at *p* < 0.05.

Microbial alpha and beta diversities in the cecum were analyzed to assess the impact of different breeding methods (Figure 2). Alpha diversity was assessed utilizing the diversity indices (Simpson and Shannon), community richness (Chao1 and ACE) and sequencing depth (Goods_coverage). The Goods_coverage values approached 100% for all samples, displaying comprehensive sequencing coverage (Figure 2). The Chao1 and ACE indices in the cecum ranged from 528 to 655 and 552 to 679, respectively, while the Simpson and Shannon indices ranged from 0.901 to 0.907 and 5.33 to 5.69. Notably, Group B exhibited the highest value for Chao1, ACE and Shannon indices. However, no statistically significant differences were observed in alpha diversity among the three breeding methods. Unweighted beta diversity of Group C was significantly higher than that of groups A and B (*p* < 0.05), while no statistically significant differences were observed in weighted beta diversity.

### 3.2. Microbial Community Structure Analysis

**Figure 3:**
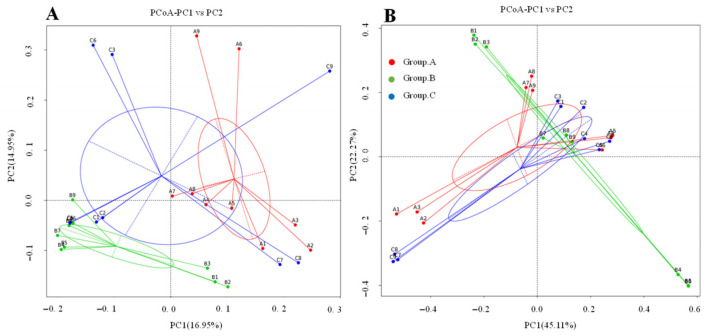
(**A**,**B**) indicates the PCoA map on the basis of the unweighted and weighted UniFrac distance, respectively. Each point on the map represents one sample. The distance between the two points represents the difference in the cecum microbiota of the different samples. A1 to A9 represent the sample in Group A. The same as B1 to B9, C1 to C9.

Both the weighted and the unweighted PCoA plots revealed the distinct clustering patterns among the three breeding methods, with sample proximity reflecting similarity in species composition (Figure 3). Under the unweighted UniFrac distance, Group B samples showed minimal overlap with groups A and C, whereas the weighted UniFrac analysis revealed greater overlap among all groups. The results of multi-response permutation procedures (MRPP) further confirmed significant community differences, with between-group variations exceeding within-group differences (Table 2, *p* < 0.05).

### 3.3. Analysis of Relative Abundance at Phylum and Genus Levels

**Figure 4:**
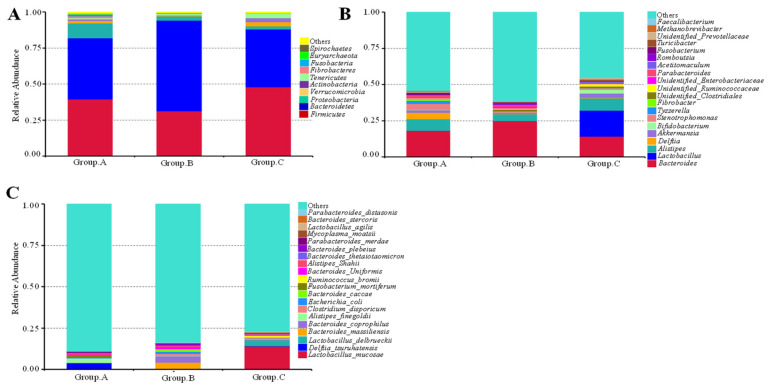
The relative abundance of the cecum microbial community at the phylum (**A**), genus (**B**), and species (**C**) levels.

**Figure 5:**
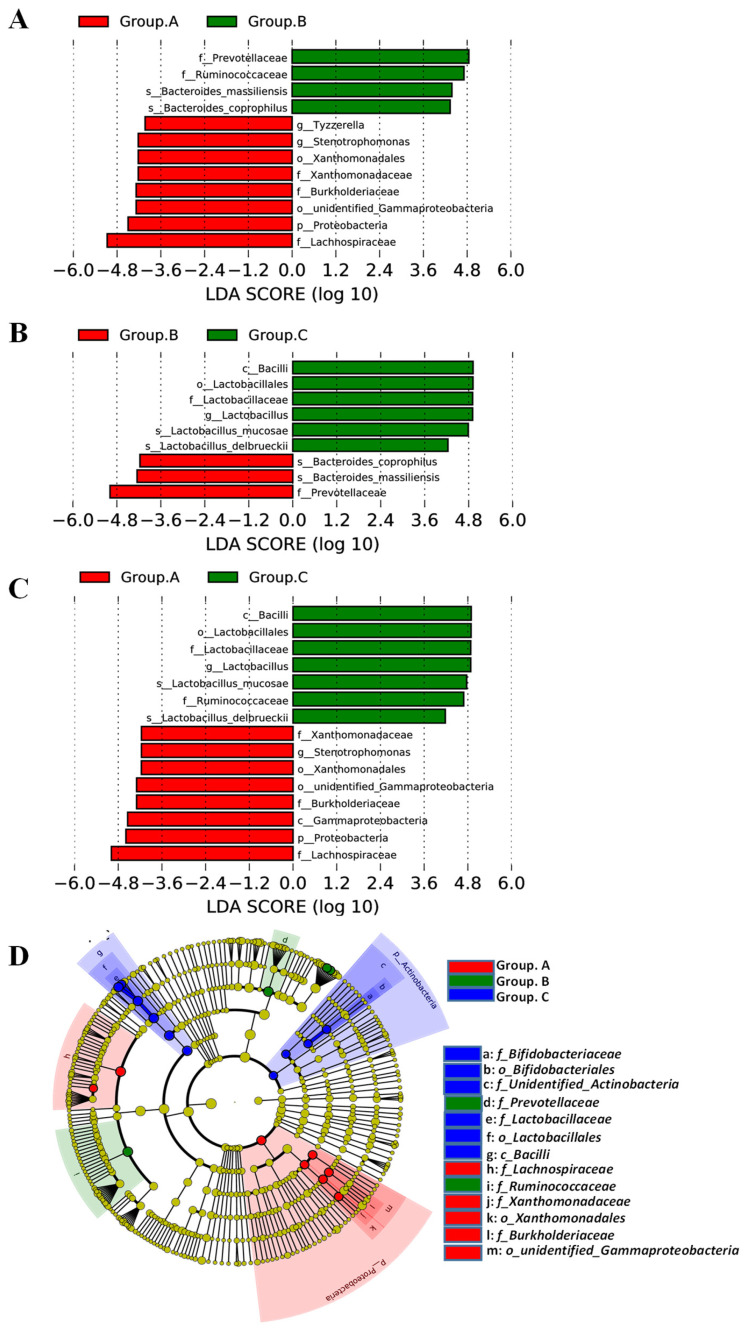
LDA value distribution histogram (**A**–**C**) and evolutionary branching diagram (**D**) based on OTUs by LEfSe analysis. LDA value > 4 was considered statistically significant. The circles with different colors in the cladogram from the inside to the outside indicate phylum, class, order, family, and genus levels, respectively.

The relative percentages of predominant microbial taxa at the phylum, genus and species levels were assessed under different breeding methods. The top 10 phyla, 20 genera and species identified in cecal microbiota were presented in Figure 4. At the phylum level, the cecal microbiota of YBGs in groups A and B were dominated, in descending order, by *Bacteroidetes*, *Firmicutes*, and *Proteobacteria* (Figure 4A). In Group C, the microbiota was primarily composed of *Firmicutes* and *Bacteroidetes*. At the genus level, taxa with relative abundances exceeding 1% in Group A included *Bacteroides*, *Alistipes*, *Delftia*, *Stenotrophomonas*, *Tyzzerella*, *Akkermansia*, *Parabacteroides*, *Fusobacterium*, and *Fibrobacter* (Figure 4B). In Group B, the predominant genera were *Bacteroides*, *Alistipes*, *unidentified_Clostridiales*, *Romboutsia*, *unidentified_Enterobacteriaceae*, and *Parabacteroides*. In Group C, genera with relative abundances above 1% comprised *Lactobacillus*, *Bacterioides*, *Alistipes*, *Akkermansia*, *Bifidobacterium*, *undidentified_Ruminococcaceae*, *Turicibacter*, *Stenotrophomonas*, and *unidentified_Clostridiales*. At the species level, taxa with elative abundances exceeding 1% in Group A included *Delftia_tsuruhatensis*, *Alistipes_finegoldii*, and *Fusobacterium_mortiferum*. In Group B, the predominant genera were *Bacteroides_massiliensis*, *Bacteroides_coprophilus*, *Clostridium_disporicum*, *Escherichia_coli*, *Bacteroides_caccae*, and *Bacteroides_uniformis*. In Group C, the predominant genera were *Lactobacillus_mucosae*, *Lactobacillus_delbrueckii*, and *Ruminococcus_bromii*. LEfSe analysis results were presented in Figure 5. Comparison between Group A and Group B showed that *Proteobacteria* at the phylum level, and *Tyzzerella* and *Stenotrophomonas* at the genus level were the dominant floras in Group A (Figure 5A). Comparisons between Group A vs. Group C and Group B vs. Group C indicated that *Lactobacillus* at the genus level was the dominant flora in Group C.

The relative abundance of *Proteobacteria* was significantly higher in Group A compared with groups B and C (Table 3, *p* < 0.05). Other phyla, including *Verrucomicrobia*, *Actinobacteria*, *Tenericutes*, *Fibrobacteres*, *Fusobacteria*, *Euryarchaeota*, and *Spirochaetes*, were present at low abundances (each <5%). Notably, *Stenotrophomonas*, *Tyzzerella*, *Parabacteroides, and Fusobacterium* in Group A; *unidentified_Clostridiales* and *Romboutsia* in Group B; and *Lactobacillus*, *unidentified_Ruminococcaceae*, and *Turicibacter* in Group C, showed significantly higher relative abundances compared with the other groups at the genus level (Table 3, *p* < 0.05). *Alistipes_finegoldii* in Group A, *Bacteroides_massiliensis* and *Bacteroides_coprophilus* in Group B, and *Lactobacillus_mucosae* and *Lactobacillus_delbrueckii* in Group C, showed significantly higher relative abundances compared with the other groups at the species level (Table 3, *p* < 0.05).

### 3.4. Functional Prediction Analysis

**Figure 6:**
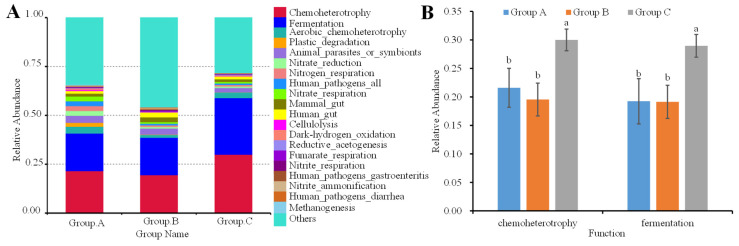
Bacterial functional prediction analysis using FAPROTAX(v1.2). (**A**) presents the top 20 functional prediction; (**B**) demonstrated the differences of top two functional prediction. Different lowercases represent the significant differences at the 0.05 level.

Functional prediction analysis revealed conserved functional pathway profiles across the three groups. The metabolic function of cecal microbiota was assessed using FAPROTAX. At category level 1, the most differentially represented functional pathways were primarily associated with chemoheterotrophy and fermentation (Figure 6A). Comparative analysis demonstrated that both pathways were significantly enriched in Group C relative to other groups (Figure 6B).

### 3.5. Production Traits

The results exhibited a significant effect of the three breeding methods on total weight gain of YBGs (Table 4). Specifically, Group A exhibited the highest TWG, followed by Group B, with statistically significant differences observed between the groups (*p* < 0.05). While carcass rate did not show significant variation among the three groups (*p* > 0.05).

### 3.6. Correlation Analysis of Cecal Microorganism and Production Traits

**Figure 7:**
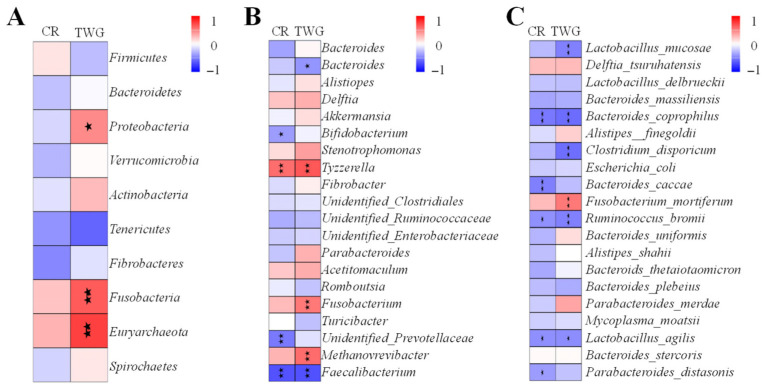
Correlation analysis between cecal microorganisms and production traits. (**A**) Phylum level; (**B**) genus level. (**C**) Species-level “
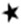
” and “
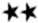
” represent the significant correlation at *p* < 0.05 and *p* < 0.01, respectively.

Several significant correlations were observed between cecal microorganisms and production traits at both phylum and genus levels (*p* < 0.05, Figure 7). At the phylum level, TWG was significantly and positively correlated with *Proteobacteria*, *Fusobacteria* and *Euryarchaeota* (*p* < 0.05, Figure 7A). At the genus level, significant positive correlations were identified between CR and *Tyzzerella*, as well as TWG and *Tyzzerella*, *Fusobacterium*, and *Methanoverevibacter* (*p* < 0.05, Figure 7B). In contrast, significantly negative correlations were observed between CR and *Bifidobacterium*, *unidentified_Prevotellaceae*, *Faecalibacterium*, and similarly between TWG and *Bacteroides* and *Faecalibacterium*. At the species level, CR was significantly and negatively correlated with *Bacteroides_coprophilus*, *Bacteroides_caccae*, *Ruminococcus_bromii*, *Lactobacillus_agilis*, and *Parabacteroides_distasonis*, as well as TWG and *Lactobacillus_mucosae*, *Bacteroides_coprophilus*, *Clostridium_disporicum*, *Ruminococcus_bromii*, and *Lactobacillus_agilis*. In contrast, significant positive correlations were only observed between TWG and *Fusobacterium_mortiferum*.

## 4. Discussion

YBGs are a local breed in China. This study aimed to characterize the cecal microflora under different breeding methods using 16S rRNA gene sequencing. The results revealed that *Bacteroidetes*, *Firmicutes* and *Proteobacteria* were the dominant phyla in the cecal microbiome of YBGs, accounting for 48.5%, 39.6% and 5.1% in all samples, respectively. The predominant phyla suggested that digestion and absorption in YBGs were primarily mediated by *Bacteroidetes* and *Firmicutes*, which is consistent with previous findings in ruminants [20,26,27]. *Bacteroidetes* are closely related to the conversion of DNA, proteins, lipids, and other organic substances, and participate in the metabolism of saccharides, bile acids and steroids [28,29]. *Firmicutes* are the primary fiber-degrading bacteria that decompose fibers, facilitating cellulose decomposition [30] and polysaccharide fermentation [29]. The high abundances of *Bacteroidetes* and *Firmicutes* in the gut environment might be linked to the host’s energy and nutritional demands [31]. *Proteobacteria* displayed diverse metabolic functions that help to meet the host’s high energy and nutrient demands [32], while also serving as a potential diagnostic signature of dysbiosis and risk of disease [33].

Previous studies had demonstrated that breeding methods significantly influenced growth performance, metabolism, physiology, gastrointestinal fermentation and microbiota in various organisms [3,34]. As a crucial fermentation site in ruminants, the cecum plays a vital role in digesting residual fiber components that are fermented incompletely in the rumen. These undigested substrates were further metabolized by the cecal microbiome to produce volatile fatty acids, which are subsequently absorbed and utilized in the cecum by the host [35]. While the effects of breeding methods on cecal microbiota have been studied in Tibetan pigs [36] and Huang-huai sheep [34], limited information is available regarding their impact on YBGs. This study addresses this knowledge gap by comparing the cecal microbiome structure and composition between stall-fed and grazing YBGs. This result revealed that breeding methods induced significant alterations in the cecal microbial community, highlighting their importance in ruminant nutrition and management.

Stall feeding differed from grazing, resulting in dietary changes for YBGs. Previous studies have shown that the fecal microbiota of cattle was strongly influenced by forage- and concentrate-based diets [37,38]. In this study, the total number of cecal OTUs in YBGs was higher under grazing than under stall feeding, whereas the core OTUs showed the opposite trend. These observations may be attributed to the diverse forage types available during grazing and the higher nutritional levels provided in stall feeding [35]. However, no significant differences were observed in the alpha diversity of cecal microflora in YBGs. This could be due to the stabilization of dominant bacteria after weaning [39]. Nevertheless, according to the results of MRPP, the differences between groups were significantly greater than those within groups across all three groups (*p* < 0.05). This indicated that breeding methods could influence the composition of bacteria colonizing the cecal tract of goats [34].

Breeding methods and the feed types have been shown to alter the abundances of *Bacteroidetes*, *Firmicutes* and *Proteobacteria* in many studies [26,40]. In this study, a higher relative abundance of *Firmicutes* was observed in Group C. Although not statistically significant, this finding aligns with the greater types of forage grass intake reported in YBGs of Group C, which may promote *Firmicutes* proliferation [37,38]. The highest abundance of *Bacteroidetes* was found in YBGs of Group B, which contrasts with the results of Cui et al. [26], who reported significantly more *Bacteroidetes* in native-pasture-fed sheep compared to oat-hay-fed sheep. This discrepancy may be explained by the higher carbohydrate concentration in the roughage provided to Group B compared to the other groups. Additionally, *Proteobacteria* reached their highest abundance in Group A, which was fed a total mixed ration (TMR). This observation is consistent with the findings of Liu et al. [41], who also reported that *Proteobacteria* were more abundant in the rumen of goats fed a complete feed diet compared to those fed only forage.

In addition, several predominant genera belonging to the phylum *Firmicutes*, including *Lactobacillus*, *unidentified_Ruminococcaceae*, and *Turicibacter*, were found at significantly higher levels in Group C compared to the other groups. *Lactobacillus* was recognized as a beneficial intestinal bacterium with advantageous biological properties such as growth promotion, immune enhancement, maintenance of micro-ecological balance, and prevention of pathogenic invasion [32,42]. Compared to YBGs in groups A and B, YBGs in Group C fed forages with longer particle sizes during grazing, which may have contributed to the elevated Lactobacillus levels [43]. This effect could be attributed to dietary non-starch polysaccharides (such as fibers), which promote carbohydrate fermentation and enhance microbial activity in the hindgut [44]. Longer forage might also provide slowly fermentable carbohydrates that support the proliferation of beneficial bacteria [45]. *Ruminococcaceae* could enrich genes encoding endo-1, 4-β-xylanase and cellulose, which are critical for degrading cellulose and hemicellulose in plant material [46]. Their higher abundance in forage-fed YBGs of Group C may positively regulate immune function and improve the intestinal environment [47]. *Turicibacter* could modify host bile acids and lipid metabolism [48]. Previous studies reported that its relative abundance often negatively correlates with dietary fat intake [49] and adiposity [50]. In line with this, YBGs under grazing conditions, which exhibited the lowest total weight gain in this study, showed the highest relative abundance of *Turicibacter* in the present study. The genus *Tyzzzerella*, also within *Firmicutes*, has been associated with unhealthy diets and Crohn’s disease [51]. In this study, YBGs in Group A displayed the highest relative abundances of this genus, suggesting that long-term TMR feeding may not be ideal. Clostridiales play an important role in the digestion of poly- and oligosaccharides [52]. Romboutsia, a beneficial bacterium, could produce short-chain fatty acids, especially butyric acid, and help modulate intestinal inflammation and oxidative stress through immunomodulatory mechanisms [53,54]. In Group B, YBGs were fed roughage with higher carbohydrate content, which may have stimulated the increase in *unidentified_Clostridiales* and *Romboutsia*, thereby promoting immune -microbial homeostasis [54,55].

The genus of *Stenotrophomonas*, belonging to *Proteobacteria*, is known to trigger the release of inflammatory cytokines and contribute to mucosal damage in colitis [8]. Consistent with the findings of Liu et al. [8], who reported that a high-grain diet elevated the proportion of *Stenotrophomonas* in the small intestine compared to a hay-based diet, the present study also observed this pattern. Among the three breeding methods, TMR-fed YBGs in Group A showed the highest relative abundances of *Stenotrophomonas*, suggesting that prolonged high-nutrition TMR feeding may pose adverse health effects in YBGs. In contrast, the genus *Parabacteroides*, belonging to the phylum *Bacteroidetes*, was associated with enhanced intestinal integrity and reduced inflammation [56]. This genus was most abundant in stall-fed YBGs of Group A, indicating its potential role in supporting gut health under confined feeding conditions. Additionally, the genus *Fusobacterium*, belonging to the phylum *Fusobacteria*, was involved in fermenting carbohydrates and proteins for energy production. Previous research has shown that high-grain-fed geese exhibit a greater increase in *Fusobacterium* compared to grass-fed geese [57]. Similarly, TMR-fed YBGs demonstrated the highest relative abundances of *Fusobacterium*, further supporting the influence of dietary composition on the proliferation of this bacterial genus in this study.

Changes in the cecal microflora can reshape its functions. In the cecum of YBGs, chemoheterotrophy and fermentation were highly enriched, indicating that the majority of microorganisms present play a crucial role in decomposing organic matter for both the host and their own metabolic needs [58]. This finding aligns with the observed relative abundance of *Bacteroidetes* and *Firmicutes*. Furthermore, chemoheterotrophy and fermentation functions were most prominent in YBGs from Group C, suggesting that dietary non-starch polysaccharides (e.g., fibers) promote carbohydrate fermentation and enhance microbial activity in the distal intestinal tract [44]. However, further research is needed to describe the specific functional pathway differences among different microbial groups.

Different breeding methods could lead to varying degrees of difference in livestock feed utilization and absorption. Previous studies have indicated that stall feeding often results in better production performance compared to grazing [5,34], a finding approved by this study. Specifically, both TWG and CR of YBGs were higher in stall feeding of groups A and B than in grazing of Group C. TMR feeding has gained wider acceptance among researchers recently [10]. In this study, YBGs fed TMR in Group A showed greater TWG and CR than Group B, which received concentrate and roughage separately. However, Arbaoui et al. [59] found no significant differences in TWG and CR between TMR feeding and separate concentrate and roughage feeding, even though TMR was observed to reduce rumen pH, indicating a potentially higher risk of rumen acidosis.

Based on the correlation analysis of the relationship between cecal microorganisms and production traits, it was found that TWG was positively correlated with *Proteobacteria*, *Fusobacteria*, and *Euryarchaeota* at the phylum level, a result consistent with the findings reported by Li et al. [60]. *Fusobacterium*, which can ferment carbohydrates and proteins to produce energy [57], also showed a positive correlation with TWG in this study. *Methanobrevibacter* was positively correlated with TWG and may contribute to weight regulation by influencing food digestion and calorie absorption [60]. Additionally, it has been reported that lower levels of *Bacteroides* are beneficial for fat deposition [61], which aligns with the negative correlation observed between *Bacteroides* and TWG in this study. Thus, it has been demonstrated that cecal microbiota contributes to the production traits of YBGs.

## 5. Conclusions

In conclusion, this study examined the effects of different breeding methods on the cecal microbiota of YBGs. The results showed that the cecal microbiota were primarily composed of *Firmicutes*, *Bacteroidetes*, and *Proteobacteria* at the phylum level. Additionally, breeding methods were found to partially influence changes in the relative abundances of microorganisms at both the phylum and genus levels. Significant differences among the three groups were observed in the relative abundance of *Proteobacteria* at the phylum level, in the relative abundances of *Lactobacillus*, *Stenotrophomonas*, *Tyzzerella*, *unidentified Clostridiales*, *unidentified Ruminococcaceae*, *Parabacteroides*, *Romboutsia*, *Fusobacterium*, and *Turicibacter* at the genus level, as well as *Alistipes_finegoldii*, *Bacteroides_massiliensis*, *Bacteroides_coprophilus*, *Lactobacillus_mucosae*, and *Lactobacillus_delbrueckii* at the species level. Furthermore, TWG and CR were highest in Group A, higher in Group B, and lowest in Group C. Correlation analysis revealed that *Proteobacteria*, *Fusobacteria*, and *Euryarchaeota* at the phylum level, and *Tyzzerella*, *Fusobacterium*, and *Methanobrevibacter* at the genus level were positively correlated with TWG. In contrast, *Bacteroides* and *Faecalibacterium* showed a negative correlation with TWG. Therefore, breeding methods can alter the cecal microbiota of YBGs, thereby regulating their production traits.

## Figures and Tables

**Table 1:** The main nutrient contents of TMR, concentrate, roughage and supplementary fodder %.

Fodder	Crude Fat	Carbohydrate	Crude Protein	Moisture
TMR	12.00	12.00	18.00	14.00
Concentrate	16.01	4.21	12.17	16.40
Roughage	1.70	45.30	11.60	14.50
Supplementary fodder	1.30	73.30	12.2	15.50

The nutrient contents of the dairy diet are the same as those of Yang et al. [1].

**Table 2:** Analysis of differences between MRPP groups.

Group	A-Value	Observed-Delta	Expected-Delta	Significance Value
A-B	0.1474	0.6728	0.7891	0.001
A-C	0.1095	0.7155	0.8034	0.001
B-C	0.1087	0.6981	0.7833	0.001

The A-value (>0) represents that the difference between groups is greater than the difference within groups. The smaller observed delta represents the smaller difference within the group. The larger expected delta indicates a larger difference between groups. Significance < 0.05 indicates significant differences.

**Table 3:** Effects of breeding methods on relative abundances of dominant phyla (>5%) and predominant genera and species (>1%) of cecal microorganisms of YBGs.

Taxa of Microorganism		Group A	Group B	Group C
Phylum level				
	*Firmicutes*	39.45 ± 8.18 a	31.32 ± 4.70 a	47.87 ± 10.23 a
	*Bacteroidetes*	42.38 ± 10.70 a	62.91 ± 4.94 a	40.11 ± 9.94 a
	*Proteobacteria*	10.40 ± 4.29 a	2.93 ± 0.84 b	2.09 ± 0.42 b
Genus level				
	*Bacteroides*	18.18 ± 4.85 a	24.89 ± 8.62 a	14.05 ± 4.18 a
	*Lactobacillus*	0.03 ± 0.01 b	0.03 ± 0.01 b	18.03 ± 9.21 a
	*Alistipes*	8.03 ± 2.11 a	4.57 ± 0.51 a	8.52 ± 3.64 a
	*Delftia*	4.44 ± 2.71 a	0.28 ± 0.08 a	0.29 ± 0.11 a
	*Akkermansia*	1.73 ± 1.17 a	0.81 ± 0.51 a	2.98 ± 1.90 a
	*Bifidobacterium*	0.07 ± 0.03 a	0.01 ± 0.01 a	2.54 ± 1.66 a
	*Stenotrophomonas*	4.28 ± 1.58 a	0.55 ± 0.01 b	1.12 ± 0.28 b
	*Tyzzerella*	2.11 ± 0.93 a	0.07 ± 0.02 b	0.10 ± 0.03 b
	*Fibrobacter*	1.12 ± 0.69 a	0.26 ± 0.13 a	0.07 ± 0.04 a
	*unidentified_Clostridiales*	0.16 ± 0.02 b	1.57 ± 0.62 a	1.05 ± 0.49 ab
	*unidentified_Ruminococcaceae*	0.34 ± 0.11 b	0.55 ± 0.18 b	1.73 ± 0.67 a
	*unidentified_Enterobacteriaceae*	0.38 ± 0.16 a	1.20 ± 0.66 a	0.29 ± 0.15 a
	*Parabacteroides*	1.53 ± 0.51 a	1.20 ± 0.49 ab	0.14 ± 0.06 b
	*Romboutsia*	0.25 ± 0.06 b	1.52 ± 0.43 a	0.38 ± 0.07 b
	*Fusobacterium*	1.28 ± 0.51 a	0.00 ± 0.00 b	0.01 ± 0.00 b
	*Turicibacter*	0.27 ± 0.11 b	0.59 ± 0.10 ab	1.30 ± 0.55 a
Species level				
	*Lactobacillus_mucosae*	0.000 ± 0.000 b	0.000 ± 0.000 b	0.140 ± 0.223 a
	*Delftia_tsuruhatensis*	0.039 ± 0.073 a	0.002 ± 0.002 a	0.002 ± 0.001 a
	*Lactobacillus_delbrueckii*	0.000 ± 0.000 b	0.000 ± 0.000 b	0.035 ± 0.058 a
	*Bacteroides_massiliensis*	0.000 ± 0.000 b	0.040 ± 0.058 a	0.001 ± 0.000 b
	*Bacteroides_coprophilus*	0.000 ± 0.000 b	0.037 ± 0.043 a	0.008 ± 0.007 b
	*Alistipes_finegoldii*	0.029 ± 0.042 a	0.003 ± 0.003 b	0.001 ± 0.001 b

The lowercase letters represent the significant difference in three breeding methods (*p* < 0.05).

**Table 4:** The production traits of YBGs under three breeding methods.

Traits	Group A	Group B	Group C	*p*-Value
Total weight gain (kg)	12.36 ± 0.60 a	8.96 ± 0.28 b	6.67 ± 0.41 c	<0.001
Carcass rate (%)	44.34 ± 1.14	43.69 ± 3.20	42.10 ± 1.27	0.096

Different lowercase letters represent the significant difference in the same row at *p* < 0.05.

## Data Availability

The datasets supporting the findings of this study are available in an online repository. The repository names and accession numbers are provided below: https://www.ncbi.nlm.nih.gov/sra/PRJNA1405800 (accessed on 1 January 2026).
